# Exploring the Absorption Spectra of an Ultra-Wideband Metamaterial Absorber in the Visible and Near-Infrared Regions

**DOI:** 10.3390/ma15207160

**Published:** 2022-10-14

**Authors:** Marwa M. Tharwat, Abdulaziz R. Alsulami, Amr M. Mahros

**Affiliations:** 1 Department of Electrical Engineering, King Abdulaziz University, Jeddah 21589, Saudi Arabia; 2 Department of Physics, University of Jeddah, Jeddah 21432, Saudi Arabia; 3 Department of Engineering Physics, Alexandria University, Alexandria 21544, Egypt

**Keywords:** optical absorption, plasmonics, metamaterial, FDTD

## Abstract

This paper investigates the absorption spectra of a plasmonic metamaterial absorber in the visible and near-infrared regimes by utilizing a metal-dielectric-metal (MDM) functional stack. A periodic metal-dielectric cap is introduced on top of a metallic substrate to excite surface plasmon modes. The shape of this cap and the glass coating modifies the absorbance bandwidth. Although the circular cap exhibits less broadening in the absorbance than the square one, the circular cap’s glass coating boosts the bandwidth’s expansion in the near-infrared region to about 1.65 µm. In the visible and near-infrared regimes, absorption bandwidth and spectral ratio can be tailored by modifying four distinct structural parameters. The finding shows that one can achieve an ultra-broad bandwidth that extends from 0.3 µm to 1.65 µm at 90% absorbance. The thickness of the top titanium layer, the silicon dioxide spacer thickness, the Ti-SiO_2_ cap diameter, and the sliver substrate pitch are selected to be 20 nm, 60 nm, 215 nm, and 235 nm, respectively. Furthermore, the influence of using various metals on absorption spectra has been explored in the visible and near-infrared regimes. The d metals considered for the top layer are titanium, nickel, chromium, silver, copper, gold, aluminum, and gold.

## 1. Introduction

In the recent decade, plasmonic cells have drawn attention as the best provenance that absorbs light through spreading and scattering by the highly exciting surface plasmons. Metamaterial absorber plays an essential role in many applications such as solar cells, optical devices, sensitive sensors, photovoltaic, thermal emitters, nanoantennas, optical switches, photodiodes, filters, and modulators [1,2,3,4,5,6,7]. Various techniques have been used to enhance the light absorption bandwidth, such as multilayer structures and cylinder and hole arrays [8,9,10,11,12,13]. On the other hand, several problems appear, such as the need for noble metals and how to produce broad bandwidth.

Due to simplicity and efficiency, investigating metal-dielectric-metal nanostructure absorbers has attracted many researchers to carry out considerable theoretical and experimental work about these structures [14]. Regrettably, these attempts had suffered a lack of wide absorption bandwidth, which is inadequate in some applications [15]. The MDM absorber is structured from three functional layers to boost the absorption bandwidth and spectral ratio. A periodic metal-dielectric cap is used to excite surface plasmon modes. This cap is introduced on top of a metallic substrate to cancel the transmittance [16].

Two principal tactics, the multi-sized and multilayered metamaterial perfect absorbers, are employed to increase absorption peaks and guarantee proper broadband bandwidth. An array of multi-sized plasmonic strip antenna has been constructed to design a broadband light absorber in the infrared regime [17]. Multilayered metallic dielectric quadrangular frustum pyramids have been presented to achieve a perfect absorber. Such techniques are complicated to be implemented experimentally due to technical difficulties or large unit size. Multilayer plasmonic metamaterial structures consist of a top metallic cap followed by a dielectric spacer and a perfect metallic reflector [12]. Ag-Al_2_O_3_-Ag was introduced as a periodic plasmonic structure to achieve absorption in a narrow bandwidth, 50 nm, of the visible region. Another absorber based on Ti-SiO_2_-Al periodic plasmonic configuration extends the bandwidth to 720 nm in the visible and near-infrared regimes [15]. They used different materials for the top cap, such as Ni and Al, which produced a lower bandwidth than Ti material. A 500 nm absorption bandwidth in the visible region has been obtained using a multiband plasmonic metamaterials absorber based on Ti-SiO_2_-Al structure [18]. The dielectric-metal-dielectric-metal structure helped to achieve a 1 µm absorption bandwidth extending from the ultraviolet to near-infrared band with absorption of over 90% [18].

Metamaterial absorbers, based on an ultrathin dielectric coating applied to a reflective substrate, qualify as strong (A > 0.9) to perfect (A > 0.99) light absorption. They have many applications, such as photodetectors and photovoltaics. It should be noted that broadband absorbing has been successfully demonstrated using different configurations and structures. In general, the benchmark characteristics of a good absorber are high average absorption efficiency, low ripple percentage, and relative absorption bandwidth more significant than 50%. Densely packed nano-stars are designed and numerically investigated to provide an absorber that can achieve 91% average absorption efficiency in the wavelength range of 450–700 nm. About 80% average measured absorption over the entire visible spectrum has been performed using a broadband absorber based on a cross-trapezoid shape [8].

In this paper, the authors use a metal-dielectric-metal (MDM) functional stack to boost the absorption spectra bandwidth of a plasmonic metamaterial absorber in the visible and near-infrared regimes. We comprehensively investigate the optical spectra in terms of four featured structural parameters. The impact of modifying the thickness of both the top metallic layer and the dielectric spacer separately and concurrently on the optical absorption spectra of the MA absorber has been studied. This paper is organized as follows: the Materials and Methods section describes the proposed structure and FDTD simulation parameters. In the beginning of the Results and Discussion section, the impact of changing the cap shape and surrounding coating on the optical spectra of the MA absorber is investigated. Furthermore, the effects of independently varying four featured structural parameters are separately considered in simulations and discussion. This section also shows that the cap metallic upper layer shape is an essential parameter that significantly influences the optical absorption of the proposed broadband metamaterial absorber. Finally, the conclusions of the results obtained are provided in Section 4.

## 2. Materials and Methods

**Figure 1:**
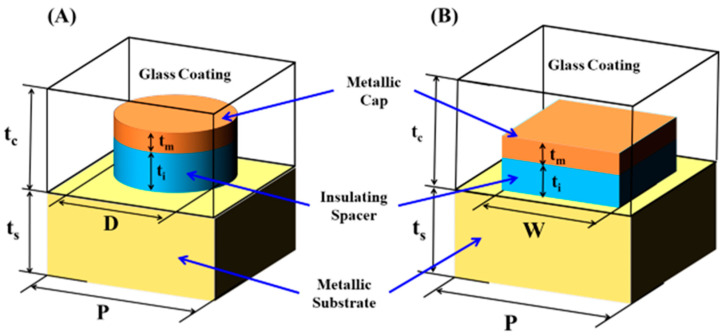
Schematic diagram of the proposed broadband MA with (**A**) circular and (**B**) square cap.

A periodic metal-dielectric cap is introduced on top of a metallic substrate to boost the absorption bandwidth and the spectral ratio. The unit cell of the proposed MA is illustrated in Figure 1 with two different schematics: (A) circular and (B) square cap. The areas of both square and circular caps are equal to guarantee fair comparison.

The cap consists of an upper titanium metallic layer with a thickness tm = 20 nm and a lower SiO_2_ insulating layer with a thickness t_i_ = 80 nm. As shown in Figure 1, (A) the circular cap with diameter D = 200 nm or (B) the square cap with width W = 177 nm is mounted on a metallic silver substrate of thickness t_s_ = 250 nm and pitch P = 250 nm. Finally, a transparent glass host medium coats the metal-dielectric cap. The refractive index of the dielectric spacer and surrounding coating were obtained from the literature [19]. The relative permittivity *ε_r_* (ω) of the dispersive metallic top layer and back reflector substrate was determined using the Lorentz–Drude model [20]:

(1) $$\varepsilon_{r}\left(\omega \right)=\varepsilon_{\infty }+\sum_{m=1}^{N}\frac{f_{m}\omega_{om}^{2}}{\omega_{om}^{2}-\omega^{2}+i\omega \Gamma_{m}}$$

where *ε*_∞_ denotes the permittivity at infinite frequency, *f_m_* is a function of position specifying the oscillator strengths, and *Γ_m_* is the damping coefficient. The incident wave frequency and the resonant frequencies are represented by *ω* and *ω_om_*, respectively. Table 1 summarizes the titanium metallic layer parameters used in Equation (1) [21].

In this work, the optical absorption spectra of the reported plasmonic MA are obtained by solving Maxwell’s equations of different materials using the FDTD algorithm. The FDTD method was performed using the electromagnetic solver OptiFDTD 16 RC simulation tool from Optiwave Inc. [21]. We modify the cap diameter, the photonic lattice pitch, the thickness of the top metallic layer, and the dielectric spacer thickness as four featured structural parameters. In addition, we investigate the effect of changing the material of both the superior metallic layer and the inferior insulating layer of the cap as two distinct material parameters. The impacts of independently varying these parameters are considered and discussed separately.

We have used a linearly polarized Gaussian modulated plan wave source with Bloch signal in the simulation with 500 THz center frequency and 375 THz full width half maximum to realize a broadband simulation in visible and near-infrared regions. The light pulse in the time domain has an offset time of 4.2 × 10^−15^ s and a half-width of 2.3 × 10^−15^ s. We have established a simulation wafer in Cartesian coordinates x, y, and z with periodic boundary conditions (PBC) in the x- and y-directions to avail the periodicity of the MA unit cell. An anisotropic perfect matching layer (PML) was used in the z-direction to serve as an absorbing boundary condition. The simulation was performed at normal incidence. Two x-y observation areas were used, 100 nm behind the source and 50 nm after the structure, to calculate the reflectance (R) and transmittance (T) spectra through the designed absorber. One can employ the electric field distribution recognized in the simulation waver to calculate the absorption per unit volume in each cell using Equation (2). Then, the absorbed power is normalized by the incident source power [5].

(2) $$P_{abs}=-0.5\;Re\;\left(\mathrm{div}\;S\right)=-0.5\;\omega \;{\left|E\right|}^{2}\;Im\;\left(\varepsilon \right)$$

where **S** denotes the Poynting vector, |**E**| is the magnitude of the electric field intensity on the selected monitor, and *ε* specifies the material dielectric constant. The incident wave frequency is represented by *ω*.

## 3. Results and Discussion

**Figure 2:**
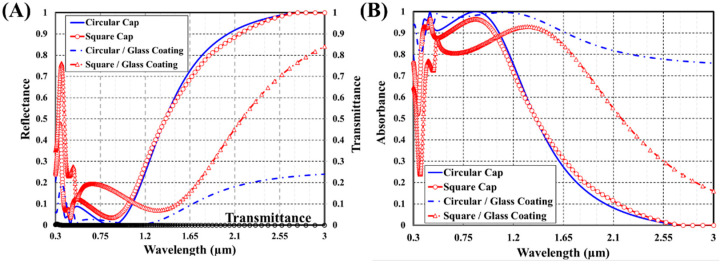
(**A**) Transmittance, reflectance, and (**B**) absorbance spectra of the proposed broadband plasmonic absorber with square and circular cap in air and glass coating.

First, the impact of changing the cap shape and surrounding coating on the optical spectra of the MA absorber has been investigated. Figure 2 presents the transmittance, reflectance, and absorbance spectra of the proposed broadband plasmonic absorber for structures with circular and square caps coated with air and glass surrounding layers throughout the wavelength window (0.3–3 µm).

As illustrated, the transmission spectrum is completely vanishing while the absorption spectrum exhibits enhanced peaks opposite to highly attenuated troughs in the reflection spectra. The thickness of the silver substrate cancels the transmittance, so the absorbance (A) spectrum can be obtained by A = 1 − R. The absorption peaks indicate less reflected optical energy due to perfect matching between the plasmonic MDM resonator and the surrounding medium. Those crests may be associated with surface plasmon resonance at metal/insulator interfaces, metallic localized plasmon resonance, and dielectric Fabry–Perot cavity resonance.

Adding an ultrathin dielectric coating to a reflective substrate enabled perfect light absorption. Light is entirely trapped due to amplitude splitting destructive interference. As the top and bottom metal layers are highly reflective, asymmetric Fabry–Perot conditions can be met. The resonance wavelength at which maximum absorption occurs for the insulator-metal stack is provided by [22,23].

(3) $$\lambda_{resonance}=2\pi n_{i}t\;\tan n_{i}\sqrt{\frac{\left(n_{m}-n_{0}\right)}{n_{0}\left(n_{i}^{2}-n_{m}n_{0}\right)}\;}\;\;\;\;$$

where *n_m_* is the refractive index of the metallic substrate, *n_i_* is the insulator refractive index, *n*_0_ is the superstrate refractive index, and *t* is the dielectric material thickness.

The titanium top layer is highly lossy. Therefore, it furnishes a low-quality factor that causes broadening. Using a glass coating layer as a cap host medium boosts the incident light absorption bandwidth. The plasmonic resonances at the metal/air interface occur at shorter wavelengths than at the metal/glass interface. One may notice that the circular cap enhances the absorbance bandwidth more than the square one. This superior enhancement can perhaps be explained by the nature of the surface plasmon waves, which are naturally surface bounded. The curved-space waveguide enables direct near-field coupling. Thus, the plasmonic beam exhibits rapid spreading on curved surfaces and will, therefore, reach the region in which the surface is curved in the opposite direction [24].

**Figure 3:**
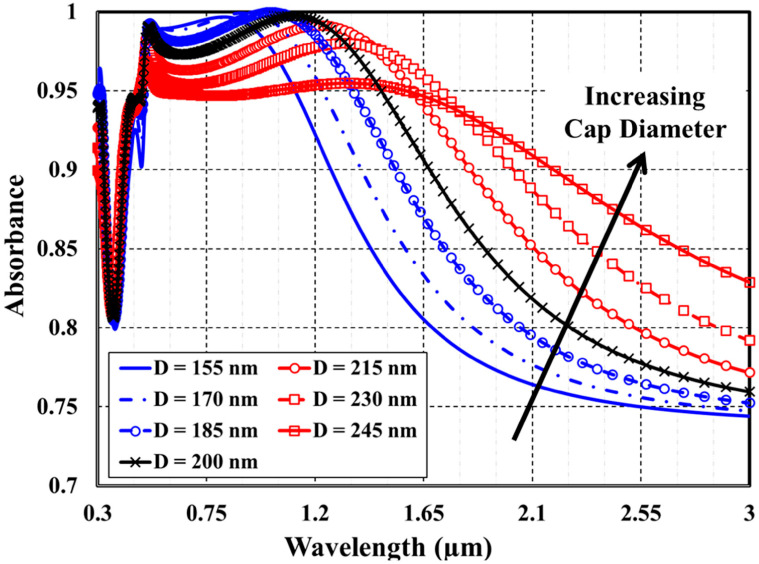
Effect of changing cap diameter on the absorbance spectra.

**Figure 4:**
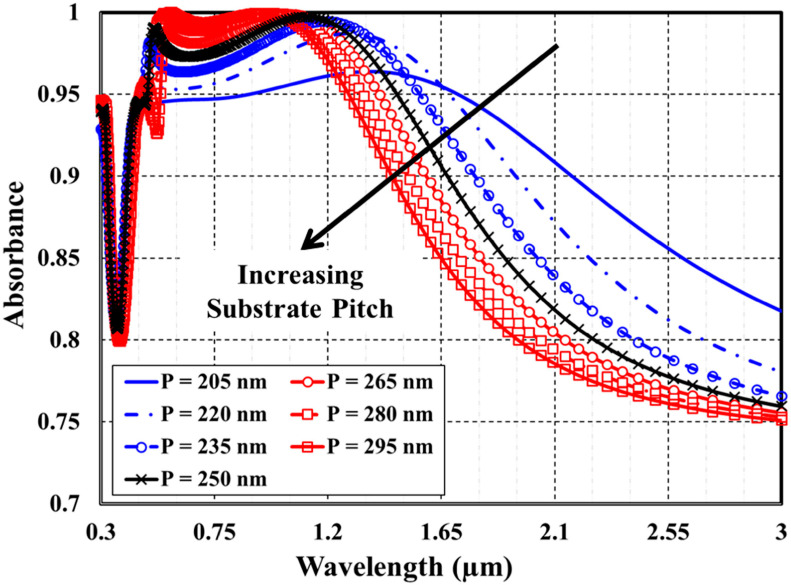
Effect of changing substrate pitch on the absorbance spectra.

Second, we have investigated the impact of modifying both the cap diameter and substrate pitch, independently and collaboratively, on the optical absorption spectra of the MA absorber. Figure 3 illustrates some examples of the absorption spectra of the designed structure at different values of the cap diameter D. The diameter has been changed within a range of 155–245 nm with a 15 nm step size. At the same time, the silver substrate pitch, substrate thickness, top titanium layer thickness, and SiO2 dielectric spacer layer thickness are kept constant at 250 nm, 200 nm, 20 nm, and 80, respectively. The impact of varying substrate pitch, in the range of 205–245 nm, on the absorption spectra of the proposed plasmonic absorber is exhibited in Figure 4. The cap diameter is kept fixed at 200 nm.

As demonstrated in Figure 3, the absorption bandwidth becomes more expansive and absorbance decreases as the cap diameter increases. In the visible region, the destructive interference of the scattered light may cause absorption quenching. In the infra-red regime, the localized surface plasmon resonance enhances the forward scattering and boosts the absorption bandwidth. An opposite behavior is noticed in Figure 4. As the photonic lattice pitch increases, the absorption bandwidth becomes more narrow and the absorbance increases.

**Figure 5:**
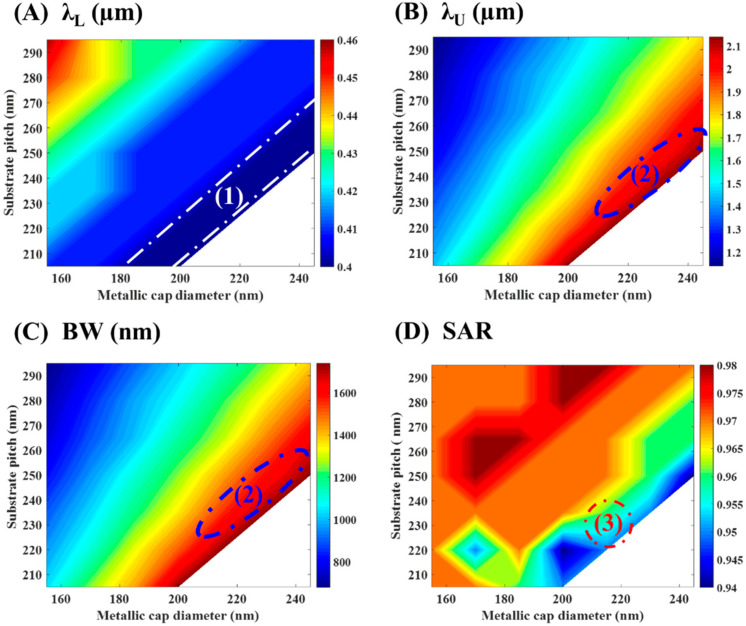
Effect of changing the cap diameter and substrate pitch on (**A**) λ_L_, (**B**) λ_U_, (**C**) BW, and (**D**) SAR. 1, 2, and 3 are regions of interest to target broad bandwidth.

Inspired by that agonistic behavior due to varying the cap diameter and substrate pitch independently, a collaborative study is presented in Figure 5.

For absorbance over 90%, the effective bandwidth *BW* is calculated as the difference between the upper wavelength *λ_U_* and the lower wavelength *λ_L_*. The average spectral absorption rate (*SAR*) is calculated numerically in that regime using Equation (4):

(4) $$SAR=\frac{\int_{\lambda_{L}}^{\lambda_{U}}A\left(\lambda \right)d\lambda }{BW}$$

The results of Figure 5A exhibit the presence of region (1), between the white dotted lines, associated with minimum *λ_L_* below 400 nm. That region is designed to target the broad bandwidth. We can select the proper values of the cap diameter D and the substrate pitch P within that region to ensure that the proposed absorber can snare optical energy efficiently in the visible part. In Figure 5B,C, the blue oval indicates the area (2) corresponding to superior absorption bandwidth and upper wavelength. Finally, at region (3) in Figure 5D, a maximum average spectral absorption rate of 95% is picked out at D ≈ 210–220 nm and P = 230–240 nm.

**Figure 6:**
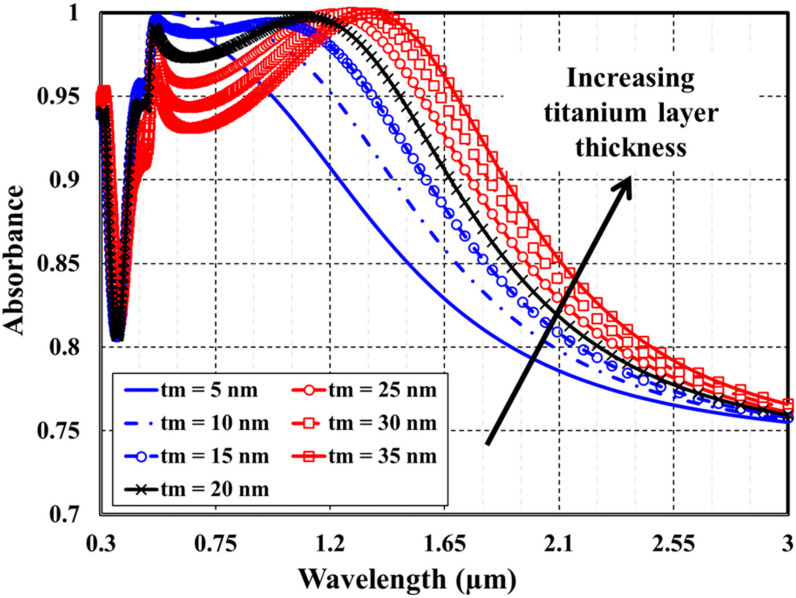
Effect of changing titanium layer thickness on the absorbance spectra.

**Figure 7:**
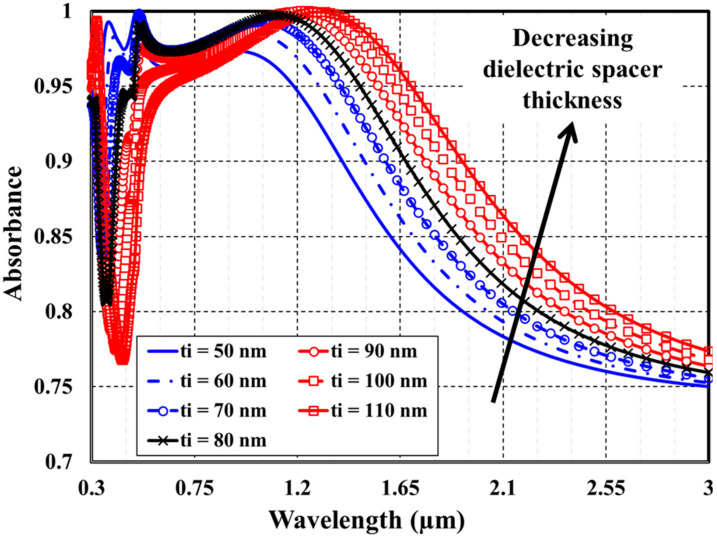
Effect of changing the dielectric spacer thickness on the absorbance spectra.

Then, to finish our investigation of the four featured structural parameters, the impact of modifying the thickness of both the top metallic layer and the dielectric spacer separately and concurrently on the optical absorption spectra of the MA absorber has been studied. Figure 6 demonstrates some samples of the absorption spectra of the proposed absorber at various values of the top metallic layer thickness t_m_. The titanium layer thickness has been modified within a range of 5–35 nm with 5 nm step size. At the same time, the silver substrate pitch, substrate thickness, cap diameter, and SiO_2_ dielectric spacer layer thickness are kept constant at 250 nm, 200 nm, 200 nm, and 80, respectively. The impact of modifying dielectric spacer thickness t_i_, in the range of 50–110 nm, on the absorption spectra of the plasmonic structure is exhibited in Figure 7. The titanium layer thickness is kept fixed at 20 nm.

**Figure 8:**
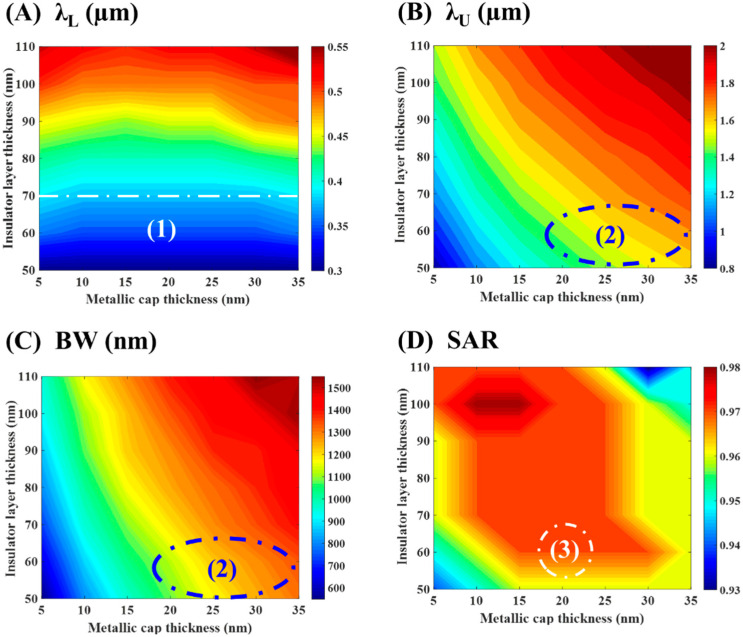
Effect of changing the titanium layer thickness and the dielectric spacer thickness on (**A**) λ_L_, (**B**) λ_U_, (**C**) BW, and (**D**) SAR. 1, 2, and 3 are regions of interest to target broad bandwidth.

As demonstrated in Figure 6 and Figure 7, the absorption bandwidth becomes more expansive and absorbance decreases as either the thickness of top metallic layer or the dielectric spacer increases. Increasing the plasmonic resonator length causes broadening of the absorption spectra. In a similar manner, Figure 8 presents a concurrent study of (A) λ_L_, (B) λ_U_, (C) BW, and (D) SAR as a function of both the thickness of top metallic layer and the dielectric spacer.

The outcomes of Figure 8A demonstrate the existence of area (1), below the white dotted line, associated with minimum *λ_L_* below 400 nm. That region is designed to target the broad bandwidth. We can choose the suitable value of the thickness of both the top metallic layer t_m_ and the dielectric spacer t_i_ within that area to ensure that the proposed absorber can trap optical energy efficiently in the visible part. Furthermore, in Figure 8B,C, the blue oval indicates the region (2) corresponding to outstanding absorption bandwidth and upper wavelength. Finally, at region (3) in Figure 8D, a maximum average spectral absorption rate of 95% is obtained at t_i_ ≈ 60–65 nm and t_m_ = 18–23 nm.

**Figure 9:**
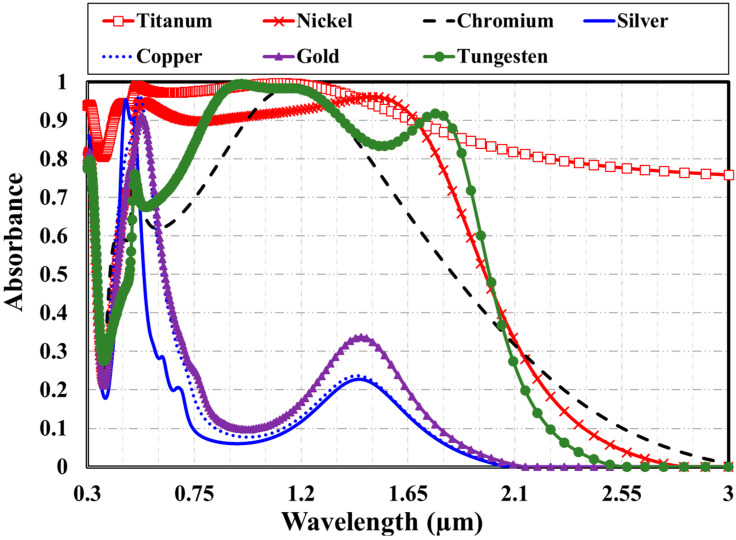
The absorbance spectra for the reported MDM plasmonic absorber with different cap top metals.

Another important parameter that may have a significant influence on the optical absorption of the proposed broadband metamaterial absorber is the type of upper metallic layer of the cap. We here consider using titanium, nickel, chromium, silver, copper, gold, and aluminum for the upper metallic layer of the cap. Figure 9 illustrates the absorbance spectra for the reported MDM plasmonic absorber with different cap top metals in both the visible and near-infrared regimes. The MDM absorber with a top layer of Cu, Au, or Ag allows a deficient absorption in the visible and near-infrared regions. Their absorbance is very high in the 450–600 nm band. On the other hand, the MDM absorber with a top layer of Ni or Ti provides a high bandwidth. The maximum absorbance is achieved when titanium metal is used as a top metal of the cap.

It is worth mentioning that the fabrication process can be done using electron-beam lithography, inductively coupled plasma deposition, metal sputtering, reactive ion etching, and chemical wet etching. The most crucial step of this fabrication process is to make the sidewalls vertical to guarantee that no metals were deposited on the sidewalls during evaporation. However, the experimentally measured resonance peaks may disappear due to the strong absorption of the adhesive layer, which prevents any surface plasmon excitation at the metal/dielectric interface. Optical responses may differ due to subtle differences in interface roughness and geometry of the nano-pattern. Recently, a practical, low-cost route to manufacture regular, highly ordered, large-area nano-coaxial structures has been demonstrated. In addition, the excellent matching between the results obtained experimentally and by using the FDTD method validates using the simulation tool [8,25,26,27].

Table 2 compares the proposed plasmonic metamaterial absorber to structures in the literature, fabricated structures, and simulated work [22,28]. Regarding the reported absorber in this paper, it is very competitive because of its higher absorption bandwidth and low ripples.

Although several types of perfect metamaterial absorbers have been demonstrated so far, here we focus on perfect absorbers based on ultrathin dielectric coating where the absorption band is broad, tunable, and insensitive to the angle of incidence. Furthermore, its structural parameters can be changed independently, precisely, and easily.

## 4. Conclusions

The absorption spectra of a plasmonic metamaterial absorber in the visible and near-infrared regimes, utilizing a metal-dielectric-metal (MDM) functional stack, have been investigated. The shape of this cap and the glass coating strongly modifies the absorbance bandwidth. Although the circular cap exhibits less broadening in the absorbance than the square one, the circular cap’s glass coating boosts the bandwidth’s expansion in the near-infrared region to about 1.65 µm. An ultra-broad bandwidth that extends from 0.35 µm to 165 µm at 90% absorbance can be tailored by modifying four distinct structural parameters. The thickness of the top titanium layer, the silicon dioxide spacer thickness, the Ti-SiO_2_ cap diameter, and the sliver substrate pitch are selected to be 20 nm, 60 nm, 215 nm, and 235 nm, respectively. Different top cap metal materials have been simulated, such as titanium, nickel, chromium, silver, copper, gold, aluminum, and gold. Titanium material provides the best performance.

## Figures and Tables

**Table 1:** Plasmonic parameters used for the titanium metallic layer.

Term	Strength	Plasma Frequency	Resonant Frequency	Damping Frequency
0	0.1480	0.110753 × 10^17^	0.000000	0.124578 × 10^15^
1	0.8990	0.110753 × 10^17^	0.118046 × 10^16^	0.345781 × 10^16^
2	0.3930	0.110753 × 10^17^	0.234724 × 10^16^	0.382547 × 10^16^
3	0.1870	0.110753 × 10^17^	0.381180 × 10^16^	0.252651 × 10^16^
4	0.0010	0.110753 × 10^17^	0.295190 × 10^17^	0.267692 × 10^16^

**Table 2:** Absorption properties of the proposed absorber and some related works.

Reference	*λ_L_* (µm)	*λ_U_* (µm)	*BW* (µm)	Amin	Amax	*SAR*
Proposed	0.4	1.65	1.25	0.90%	98%	95%
[23]			0.71		99.8	97%
[28]	0.32	0.98	0.66		97%	
